# Practical Suggestions to Young Veterinarians

**Published:** 1882-07

**Authors:** Ezra M. Hunt

**Affiliations:** Secretary New Jersey State Board of Health


### ART. XVII.—PRACTICAL SUGGESTIONS TO YOUNG VETERINARIANS: ABSTRACTS OF AN ADDRESS DELIVERED BEFORE THE GRADUATING CLASS OF COLUMBIA VETERINARY COLLEGE.

BY EZRA M. HUNT, M.D.
Secretary New Jersey State Board of Health.

Permit me, gentlemen, in closing, to say a few words to you, as students or graduates, as to how you shall best carry on this your work, and give to your profession the certification and success which it needs and which is its due.

That young man will be a success in the veterinary profession who to-night feels that he has but little more than acquired a knowledge of scope and of methods, and who resolves that he will *now* become a thorough student, and with the guide he has received *proceed* to study this science and this art for at least ten years of his life, as if he were in a part of his preparatory course. It is not worth the while of any man who is not satisfied only just to have been trained into methods, and who is not willing to be a close student for many years to come, to attempt this department. The scope with any one animal is almost equal to that which the human physician attempts on his own chosen being. I have almost felt that it would be wise to attempt the study and treatment of but one animal at a time, until at least four or five of the more important ones are taken in their turn.

It is mostly *foundation work* that you are after in such an institution as this. It is a discovery of defects, a training in methods, a habit of thoroughness, thoroughness as far as you go, that you need to seek and to secure here. Oh! the narrowness of the student who carries away with him his budget of facts and prescriptions and imagines that he has a store. Oh! the breadth of the man who just feels he has found out how to study, how to acquire, how to amass, how to dispense abroad, and beholds the boundlessness of possibilities and probabilities before him if only he is diligent, fervent, and faithful.

I know a man who built a house on a too narrow foundation. The mason concealed his four inches of blunder, and so in a year or two he had to arch, prop and patch all through the basement. That narrow foundation ought to have been seen to then and there. It is just as poor policy for you to go on accumulating superstructure and piling up knowledge unless the groundwork is right. Not only is knowledge power, but to know how to acquire knowledge is more power. Half knowledge of methods is not only, as a rule, powerless for good, but is that little learning which is a dangerous thing. The man who is ever busy to store his memory in his college course, instead of still more devoting himself to being trained in methods, is sure to be superficial and to be full of piece-meal ideas. This may make you to seem narrow in the start, and not to be a man of *general* information, but it makes you specific, and that assures increase and endurance into the end.

The habit of minute observation and introspection, and of a knowledge of details, while needed for every professional man, is especially to be commended to you. General knowledge always passes for more than it is worth in any department in which educated practitioners or experts are scarce. This easily leads to superficiality even on the part of some graduated practitioners, since general information is worth very little to practice by. Be, therefore, very close observers, even if afterward you have to study out the significance of your observation. Men even when admiring animals in general, are *not* close observers of particulars as to them. I think I may illustrate by five or six questions in your line of study, which perhaps it is not presumptious to say that all of you could not answer.

Are a cow's ears before or behind a line with the roots of the horns, and, if so, what is the usual angle? How many teeth are calves, colts, and lambs born with? What animals have eyelashes and eyebrows? Why do not cows sit down to rest the same as dogs? Why does a cow get up on her hind feet, and a horse on the front feet? Why does a sheep kick with its front feet, while mules have a well sustained notoriety for kicking with all their hinder parts, backward, upward, and onward? Accurate knowledge of the habits and functions of animals is the more needed by you, because there are many things that, if done well, must be done by yourselves instead of being, as they would be by human doctors, committed to nurses and attendants. If your medicine is to be effective in a case of active inflammation, often you must give it yourself to know that it is rightly administered with out waste. The bandaging cannot be left to others. The use of the thermometer, as I have seen it used by veterinarians, is, in the hands of three-fourths of them, of little or no diagnostic value. Yet, if used with all the minuteness of knowledge and method desirable, and tested sufficiently often, it is of great service. Close observing and accurate executing of what your knowledge and observation convinces you of is indispensable, if perfection in your art is to be acquired and the science advanced.

The stable and the cow-pen may not seem to invite to refinements of tastes. The ostler and the herdsman may not, as a rule, belong to select company; yet be assured there is such a thing as treating every man in his position with respect and of acting so as to secure his appreciation and patronage, and yet not accept the common level of those with whom he mingles.

If your mouths are to be befouled by the uncourtly oath, if the bar and the treat are the necessary concomitants of your vocation, if soiled clothing and unkempt person are to be accepted as in accord with your duties, then, I beseech you, cry out for the flesh-pots of Egypt to-night. I know of no class of men who more need to practice cleanliness of person, speech and behavior. I wish I could have each one of you take a few object lessons in neat practice from two distinguished veterinarians I have met. They know how to pick up a horse's foot secundum artem, how to move about a stable, not daintily, but properly, how to slip on and off the needed covering, how to cleanse the hands and instruments, how to be properly familiar with groom and herdsman and jockey, and yet have professional individuality of their own—in a word, how to carry their profession with them with no arrogance, with no undue reserve, and yet so that the calling is appreciated through the person. I could point you to many others I have met whose skill I know and whose attainments I have fathomed through their unkempt surroundings, who in their actual daily practice belittle their calling by their disregard of water and their careless toleration of dirt.

This whole subject of sanitary police should impress itself upon all veterinarians. Its details are matters for definite instruction as to methods.

I have met so many gentlemen in this calling, and yet have seen such contrasts of carelessness and thriftlessness, that I know you will not mistake my appreciation when I thus speak of mode and manners, or what the ancient ethics called minor morals, and commend all the details of sanitary order and cleanliness as to yourselves, your instruments, your entire equipment, armamentaries, to your personal consideration and adoption.

To you who are about to go forth to the duties and the requirements of your professional life, I am glad to-night to be able to extend the greeting hand of a sister art, and to assure you that the medical profession bids you a hearty welcome within the precincts of its high court.

In the last year we have joined hands as never before. The name of Chauveau, Touissant and Pasteur have dwelt together like brothers in unity in our hearts and upon our lips. With no flourish of trumpets, but with triumphs second only to those of Jenner, and even greater in the "boundless anticipations" which it entertains, we find our pliant fingers together at work in a common laboratory.

Etiology, Physiology, Pathology, Surgery and Practice bends the knee and turn a listening ear to the voices which are coming from the animal world. The listening, the watching, the waiting, is for the interest of all living creatures. All death, all pain, all discomfort, has very much at stake. The value of foods, the conveyance of diseases, the thrift of great industries, and great matters of social and political economy are involved. The time has come when expert knowledge must be had. In the great minglings of the nations, pestilences no longer stay in their birth-places. The isolation of a sail voyage no longer secures immunity. Rinderpest, or foot and mouth disease, or the violent forms of anthrax, may arrive in cargo in the next steamer, and be a peril to the life of man and beast over a whole continent. Appeals to humanity, to self-interest, and to self-preservation are such as cannot go unheeded. I have reason to know that, at this very time, the Government of the nation and some of the States are looking for competency in these directions. Mediocrity is seeking after more knowledge, and superiority is already in full demand. Students, exact observers, clinicians, and skilled practitioners, are wanted. Professional life, your life, if true and successful, will be arduous. These are not days of sinecure in this or the medical profession. The plod and ardor of work, of discovery, of accurate seeing and close analysis, must be yours. With its toil it has its gracious satisfactions.

The advance cannot be rapid, but it will be sure. The half century for work which opens to-night before you, opens as no half century of science has opened since the world began. Applied art is the handmaid of the science you seek, and there is a full market for its skilled products. With character as your capital, with industry as your basis of exchange, with definite outlines of your pursuit, with training in methods of acquisition, and with God's blessing upon you in a ministry of good to the creatures which he has made, you go forth bearing precious seed. Between the sowing and the reaping there be many seasons; for the plants are not animals. But keep heart, go forward, be faithful. There will be a fruitage. Science is your wand, and the skill of an effective art your sickle. Like good shepherds, be diligent to know the state of the flocks, and look well to the herds. Besides the pleasure of knowledge and the joy of one's chosen art, I doubt not the lambs will be for your clothing and the cattle for your meat, and ye shall have enough for your food; for the food of your households and for the maintenance of your maidens.

